# Silane Cross-Linked Sulfonted Poly(Ether Ketone/Ether Benzimidazole)s for Fuel Cell Applications

**DOI:** 10.3390/polym9120631

**Published:** 2017-11-23

**Authors:** Zilu Yao, Mengbing Cui, Zhenghui Zhang, Liang Wu, Tongwen Xu

**Affiliations:** CAS Key Laboratory of Soft Matter Chemistry, Collaborative Innovation Center of Chemistry for Energy Materials, School of Chemistry and Material Science, University of Science and Technology of China, Hefei 230026, China; yaozl@mail.ustc.edu.cn (Z.Y.); cuimb@mail.ustc.edu.cn (M.C.); zhangzh@mail.ustc.edu.cn (Z.Z.); liangwu8@ustc.edu.cn (L.W.)

**Keywords:** proton exchange membranes, KH-560, direct methanol fuel cells, cross-linked, densely sulfonated polymer

## Abstract

γ-(2,3-epoxypropoxy) propyltrimethoxysilane (KH-560) was incorporated in various proportions into side-chain-type sulfonated poly(ether ketone/ether benzimidazole) (SPEKEBI) as a crosslinker, to make membranes with high ion exchange capacities and excellent performance for direct methanol fuel cells (DMFCs). Systematical measurements including Fourier transform infrared (FT-IR), scanning electron microscopy-energy-dispersive and X-ray photoelectron spectroscopy (XPS) proved the complete disappearance of epoxy groups in KH-560 and the existence of Si in the membranes. The resulting membranes showed increased mechanical strength and thermal stability compared to the unmodified sulfonated poly(ether ketone/ether benzimidazole) membrane in appropriate doping amount. Meanwhile, the methanol permeability has decreased, leading to the increase of relative selectivities of SPEKEBI-x-SiO_2_ membranes. Furthermore, the H_2_/O_2_ cell performance of SPEKEBI-2.5-SiO_2_ membrane showed a much higher peak power density compared with the pure SPEKEBI memrbrane.

## 1. Introduction

With fast consumption of non-renewable energy such as coal and petroleum, proton exchange membrane (PEM) fuel cells (PEMFCs) as promising energy conversion devices have recently attracted increasing attention, due to their high energy efficiency, simplicity, quick start-up and environmental friendliness for automotive, stationary and portable applications [1,2,3]. Proton exchange membranes are known as the heart component of PEMFCs. The important function of PEMs is to conduct the electrochemically generated protons and to avoid direct contact between the fuel and oxidant [4]. Meanwhile, a fuel cell membrane should be chemically and mechanically stable in the operation environment. Sulfonated perfluoropolymers such as Nafion^®^ (Dupont) are used extensively in fuel cell applications due to their reasonable ionic conductivity, excellent strength and chemical stability. However, these materials still suffer from several disadvantages in their utility and performance, including their significant manufacturing costs, poor barrier properties to methanol and severe dependence on water [5]. Therefore, in the past decades many cost efficient and high performance polymer membranes have been attempted as alternatives for PEMs, such as acid-functionalized aromatic hydrocarbon-based materials and acid-doped polybenzimidazoles (PBIs). However, these materials also have their intrinsic limitations. For example, the acid-doped PBIs are still inferior to those perfluorinated PEMs due to three reasons: (i) the proton conductivity is insufficient; (ii) the leaching of small inorganic acid molecules; and (iii) the mechanical strength and durability are inversely proportional to the acid doping level. On the other hand, the sulfonated aromatic polymers always have a negative trade-off between proton conductivity and durability. To address these issues, it is very crucial to select polymers with exceptionally stable backbones and excellent proton conductivity. Among the various types of sulfonated aromatic polymers, sulfonated polybenzimidazoles (SPBIs) containing *N*-heterocycle units are highly rigid and exhibit excellent chemical, thermal and mechanical properties, as well as outstanding film-forming properties [6,7,8]. Although SPBIs possess some good inherent physical and chemical properties, the basic imidazole repeat units, which can form acid-base pairs with the sulfonic acid groups, will affect the proton conductivity of the membranes [9].

Generally, there are three ways to improve the proton conductivity: the first approach is to enhance the density of sulfonated hydrophilic groups [10]; the second way is to position the sulfonic acid groups on side chains grafted onto the polymer main chain to form Nafion-like structure with hydrophobic main backbones and pendant side chains with hydrophilic terminated sulfonic groups [11]; and the last method is to control the arrangement of the hydrophilic and hydrophobic segments to form multiblock copolymers [12]. In our prior work, a series of side-chain-type sulfonated poly(ether ketone/ether benzimidazole) (SPEKEBI) membranes have been synthesized by a 2-stage polycondensation process to form a series of multiblock copolymers. These polymers address both the segmented architecture and side-chain-type sulfonic acid groups. What is more, densely sulfonated polymer can be easily synthesized by raising the feed ratio of sulfonated monomer and tetramine monomer to 4:1, which is marked as SPEKEBI-4 [13]. However, despite the high proton conductivity, several problems remain still open when the sulfonation degree is too high, especially when taking into account the methanol permeability and mechanical stability. Hence, further modification of SPBIs is still desired to truly unlock their potential for fuel cell applications. The cross-linking and organic-inorganic hybridization methods are efficient for overcoming the trade-off effect by creating spatial features at the submicron- or nano-scale range [14]. Shahi et al. [15] reported the synthesis of crosslinked sulfonated poly(ether sulfone) (SPES) membranes using disulfonated 4,4′-bis(4-aminophenoxy)biphenyl-3,3′-disulfonic acid (BAPBDS) as crosslinking agent to circumvent the dilution of sulfonic groups by the added linkers. Xu et al. [16] prepared a series of covalently cross-linked sulfonated poly(imide-benzimidazole) membranes using 4,4′-bibromomethenyl diphenyl ether as a cross linker which showed enhanced mechanical properties and chemical stabilities. On the other hand, the introduction of inorganic nanoparticles into the polymer matrix is also a promising technique to improve the thermal stability and water retention capacity, as well as selective barrier properties. A variety of inorganic fillers such as silica [17,18,19,20], silver nanoparticles [21], grapheme oxide (GO) [22], protonated montmorillonite (MMT-H) [23] and metal-organic frameworks (MOFs) [24] have been explored. Na et al. [17] has employed both the cross-linking method and the in situ sol-gel method to improve the thermal and mechanical stability as well as phosphoric acid loading contents of PBI membrane. In the present work, we employed similar methods and prepared a series of densely sulfonated poly(ether ketone/ether benzimidazole) (SPEKEBI)-SiO_2_ composite membranes using γ-(2,3-epoxypropoxy) propyltrimethoxysilane (KH-560) as a crosslinker with contents of 0 (pure SPBEKEI-4), 2.5, 5, 7.5, 10 wt % in the resulting membrane, respectively. The cross-linker was introduced through a facile and general heating method by forming an amide-type linkage between epoxy silane and imidazole groups. Afterwards, hybrid membranes with silane-cross-linked network structure were achieved by in situ sol-gel process in hydrochloric acid solution. Several properties including their solubility, swelling degree, oxidative and mechanical stabilities, methanol permeability and proton conductivity, were studied in detail. Meanwhile, the influence of the SiO_2_ doping level in the membrane was discussed.

## 2. Materials and Methods

### 2.1. Materials

4,4′-oxydibenzoic acid (ODBA), 3,3′,4,4′-tetraaminobiphenyl (TABP), methane sulfonic acid and γ-(2,3-epoxypropoxy) propyltrimethoxysilane (KH-560) were used as received from Energy-chemical, Inc., Shanghai, China. P_2_O_5_, and dimethyl sulfoxide (DMSO) were obtained from Shanghai Sinopham Chemical Reagent Co. Ltd., (Shanghai, China). P_2_O_5_/CH_3_SO_3_H mixture (Eaton’s reagent), Fenton’s reagent and disodium 2,2′-di(sulfopropyloxy)biphenyl (SBP) were prepared according to the literature [25].

### 2.2. Synthesis of SPEKEBI Polymer

The preparation of densely sulfonated poly(ether ketone/ether benzimidazole) (SPEKEBI-4) was reported in our previous paper [13]. The polymer was prepared by a ‘one pot’ process as follows: in a 50 mL three-necked round bottom flask equipped with a CaCl_2_ tube, 1.291 g (5 mmol) ODBA and 0.214 g (1 mmol) TABP were firstly dissolved in 10 mL P_2_O_5_/CH_3_SO_3_H and stirred at 120 °C for 4 h under a nitrogen atmosphere. Afterwards, the temperature was cooled to 80 °C, 1.897 g (4 mmol) SBP was added to the solution, and the mixture was reacted overnight. The resulting viscous red polymer solution was poured dropwise into water. The recovered crude product was then washed with deionized water, aqueous K_2_CO_3_ and water again in sequence for several times, to obtain a beige SPEKEBI polymer.

### 2.3. Preparation of the SPEKEBI-x-SiO_2_ Membranes

In this paper, SPEKBI-x-SiO_2_ notation will be used, where x represents the weight percent of KH-560 in the polymer. A representative 5 wt % silica loading SPEKEBI-5-SiO_2_ nanocomposite was prepared as follows: 0.4 g SPEKEBI powder was dissolved in 8 mL DMSO at 80 °C to give a homogeneous solution, then 0.02 g of KH-560 was added to the SPEKEBI solution and stirred for about 4 h. Afterwards, the mixture was casted onto a flat glass substrate and heated in a vacuum oven at 60 °C for 24 h to remove the DMSO completely and then treated at 180 °C for 3 h to accomplish the reaction between SPEKEBI and KH-560. The resultant membrane was peeled off from the support and immersed in HCl solution for 24 h to complete the in situ sol-gel process, washed with water several times, and dried at vacuum oven at 25 °C overnight. In this way, a cross-linked membrane with certain content of inorganic nanoparticles was obtained. The thicknesses of the obtained membranes were in the range of 40–50 μm.

### 2.4. Characterization and Measurements

#### 2.4.1. Instruments Characterization

Fourier transform infrared (FT-IR) spectra were measured on a Bruker Vector 22 with a resolution of 2 cm^−1^. A scanning electron microscope (JEOL JSM-6700F, Tokyo, Japan), which was also equipped with an energy-dispersive X-ray spectroscopy (EDS) system, was used to observe the fracture of the membrane samples and chemical analysis. XPS data were measured on an ESCALAB 250 X-ray photoelectron spectrometer for further confirmation of the existence of Si. Differential scanning calorimetry (DSC) measurements were obtained on a PerkinElmer DSC 2C apparatuses. About 30 mg of polymer and KH-560 were put into an aluminum holder under nitrogen flux and heated at 10 °C min^−1^ from 0 to 275 °C. Thermogravimetric analysis (TGA) of the membranes was determined by a Shimadzu TGA-50H analyzer at a heating rate of 10 °C min^−1^ under nitrogen atmosphere. The mechanical properties of the membranes in a hydrated state were measured by a thermo-mechanical analyzer (DMA Q800 V20.24 Build 43, TA Instruments Inc., New Castle, DE, USA) in ambient atmosphere at a test speed of 0.5 N/min with a maximum force of 18 N. Conductivity measurements were carried out by using a four-probe conductivity cell. The membrane sample was assembled into a Teflon cell with 2 current collecting electrodes and 2 potential sensing electrodes. The assembly was then placed in deionized water and the impedance spectrum was recorded. The corresponding resistance(*R*)obtained from a Nyquist, which was measured by a standard four-electrode AC impedance techniqueonan Autolab (PGSTAT 302N, Metrohm, Herisau, Switzerland) in galvanostatic mode and with an A.C. current amplitude of 0.1 mA and a frequency range from 10^6^ to 50 Hz. In-plane conductivity (σ) was calculated according to the following equation:

(1) $$\sigma =\frac{L}{RWd},$$

where *L* is the distance between potential sensing electrodes, *W* and *d* are the width and thickness of the membrane, respectively.

Methanol permeability of the membranes was measured according to our prior work [13].

#### 2.4.2. Water Uptake and Swelling Ratio Measurements

The membranes (4 × 1 cm^2^) were dried at 30 (low temperature can avoid the distortion of the membranes) under vacuum for 48 h until the weight is always constant and these values were recorded and marked as dry weights. The membranes were then soaked into deionized water for 24 h at the desired temperatures. Afterwards, the membranes were quickly taken out, wiped by filter paper and weighed, these values were also recorded and marked as wet weights. The water uptake of the membranes was calculated by the following equation:

(2) $$WU=\frac{W_{wet}-W_{dry}}{W_{dry}}\times 100\%,$$

where *W_dry_* and *W_wet_* are the masses of dried and the corresponding water-swollen samples, respectively. Meanwhile, the swelling ratio of the membranes was calculated according to Equation (3):

(3) $$LSR=\frac{l_{wet}-l_{dry}}{l_{dry}}\times 100\%,$$

Here, *l_wet_* and *l_dry_* are the lengths of wet and dry membranes, respectively.

#### 2.4.3. Ion Exchange Capacity and Oxidative Stability

The ion-exchange capacities (IECs) were determined according to previously reported methods [13]. Dry membrane samples ware accurately weighed and converted to H^+^ form in 1.0 mol/L HCl for 48 h. Excessive HCl was washed off with water, and then the samples were immersed in 0.04 mol/L NaOH for 48 h. while IEC was determined from the decrease in NaOH through acid base titration. 

Pieces of membranes (4 × 1 cm^2^) were immersed into Fenton’s reagent (3% H_2_O_2_ containing 2 ppm FeSO_4_) at 80 °C for 1 h. The stability was evaluated from changes in the weight.

#### 2.4.4. Membrane-Electrode Assembly and Single Fuel Cell Test

The membrane-electrode assemblies (MEAs) with an active area of 12.25 cm^2^ were fabricated using a catalyst-sprayed membrane method as follows: Pt/C catalysts (60% *w/w* in metal content) were ultrasonically mixed with the Nafion ionomer (DuPont, Wilmington, DE, USA) solution to yield inks containing 20% *w/w* of ionomer and 80% *w/w* of catalyst. And then, the resulting catalyst inks were used to fabricate gas diffusion electrodes (GDEs) by hand spraying onto a larger area gas diffusion layer (GDL, Toray TGP-H-060). The metal loading in both anode and cathode was controlled to be 0.5 mg Pt/cm^−2^. The resulting GDE was correctly placed on both sides of the prepared membranes to make the membrane electrode assemblies (MEAs) in situ; no hot pressing was used.

H_2_/O_2_ fuel cell (PEMFC) benchmarking tests (75 °C) with Pt-based catalysts Pt/C catalysts (60% *w/w* in metal content) were ultrasonically mixed with the Nafion ionomer solution to yield inks containing 20% *w/w* of ionomer and 80% *w/w* of catalyst.

In the single cell performance tests, highly pure H_2_ and O_2_ were humidified at 75 °C (100% relative humidity (RH)) and fed with a flow rate 1000 cm^3^ min^−1^ with a backpressure of 0 MPa symmetrically on both sides. The cell voltage at each current density was recorded after the power output stabilized.

## 3. Results and Discussion

### 3.1. Synthesis and Characterization of SPEKEBI-x-SiO_2_

**Scheme 1:**
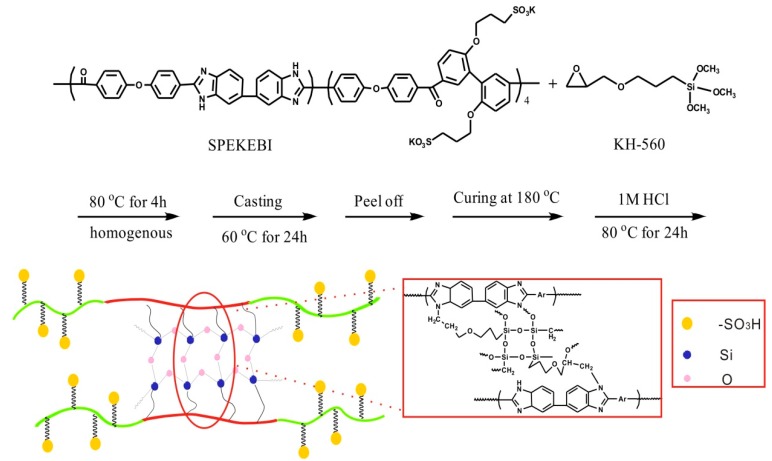
Synthesis route of sulfonated poly(ether ketone/ether benzimidazole) (SPEKEBI)-x-SiO_2_ membranes.

The synthesis of the novel silane cross-linked membranes is shown in Scheme 1. The SPEKEBI-x-SiO_2_ hybrid membranes were prepared in a convenient heating method from densely sulfonated SPEKEBI polymer and KH-560. The SPEKEBI polymer was firstly synthesized via a one-pot polycondensation reaction according to our previous paper [13]. As shown in the experiment, the reaction of SPEKEBI and KH-560 was completed by heating the membrane at 180 °C which is determined by the DSC. Then, the hybrid membranes were prepared by acid catalyzed sol-gel reaction, and nanoparticles were in situ generated within the polymer matrix. The membranes obtained through this way have uniform SiO_2_ dispersions and good organic-inorganic contact.

**Figure 1:**
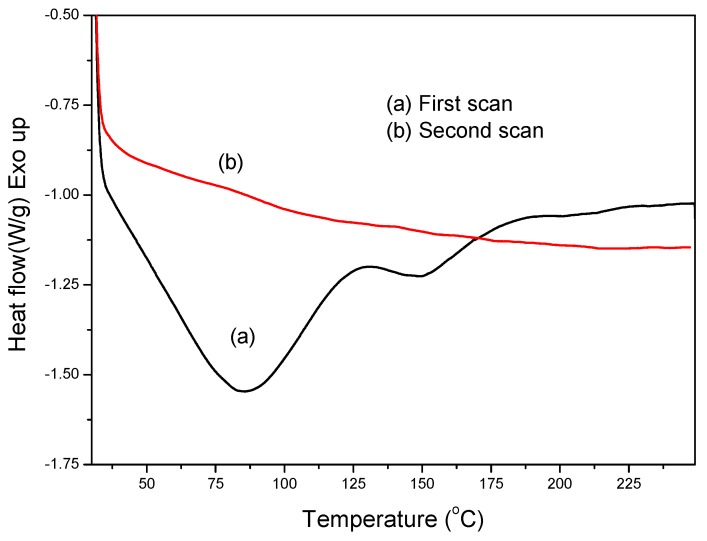
Typical differential scanning calorimetry (DSC) thermograms for dynamic scanning of sulfonated poly(ether ketone/ether benzimidazole) (SPEKEBI) with KH-560.

Differential scanning calorimetry (DSC) was employed to determine the reaction temperature between the imidazole ring of SPEKEBI and the epoxy group of KH-560 [17]. As shown in Figure 1, the mixture of the SPEKEBI and KH-560 were heated from 0 to 250 °C with a heating rate of 10 °C min^−1^. The endothermic transitions at 85 °C and 150 °C should be attributed to the melting of the KH-560 and the lipids. The exothermic peak around 180 °C is due to the etherification of KH-560 with the imidazole ring of SPEKEBI.

**Figure 2:**
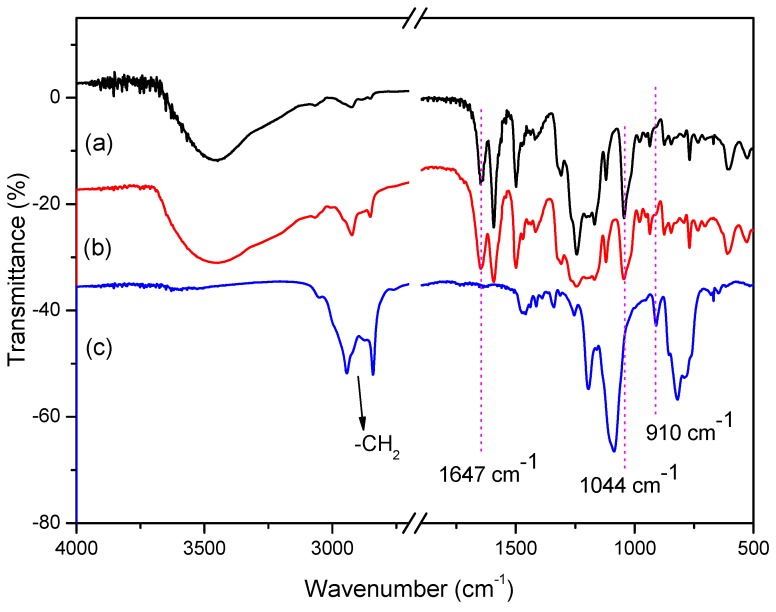
Fourier transform infrared (FT-IR) spectra of SPEKEBI (**a**), SPEKEBI/KH-560-5% (**b**) and KH-560 (**c**).

The FT-IR spectra of pure SPEKEBI, SPEKEBI-5-SiO_2_ and KH-560 are shown in Figure 2. As can be seen, the characteristic absorption band at 910 cm^−1^, which is assigned to the epoxy groups, disappeared completely in the SPEKEBI-5-SiO_2_ membrane. The characteristic absorption band at 1647 cm^−1^ was due to the C=N absorption. The strong peaks at 1047 cm^−1^ are assigned to the sulfonate groups. But the characteristic absorption for Si-O bond (usually around 1040 cm^−1^) was invisible in the cross-linked membrane, presumably due to the overlapping by the absorption of -SO_3_K groups, which lead to great difficulties to prove the existence of SiO_2_ in the membranes by FT-IR.

**Figure 3:**
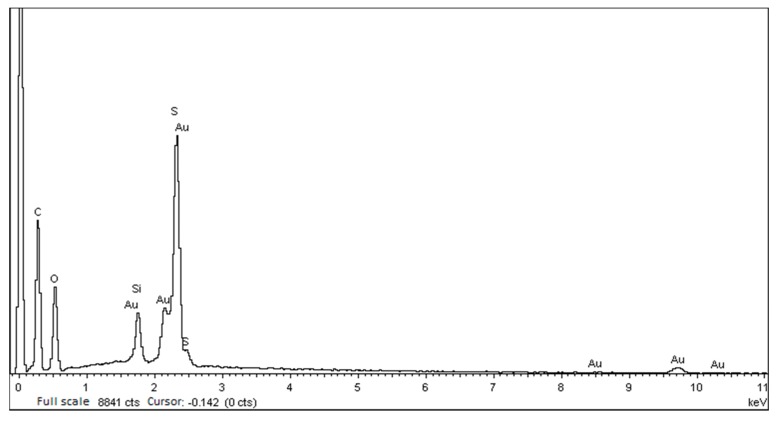
The elemental distribution cross section (EDX) spectrum of cross-sectional morphology for SPEKEBI-2.5-SiO_2_ membrane.

**Figure 4:**
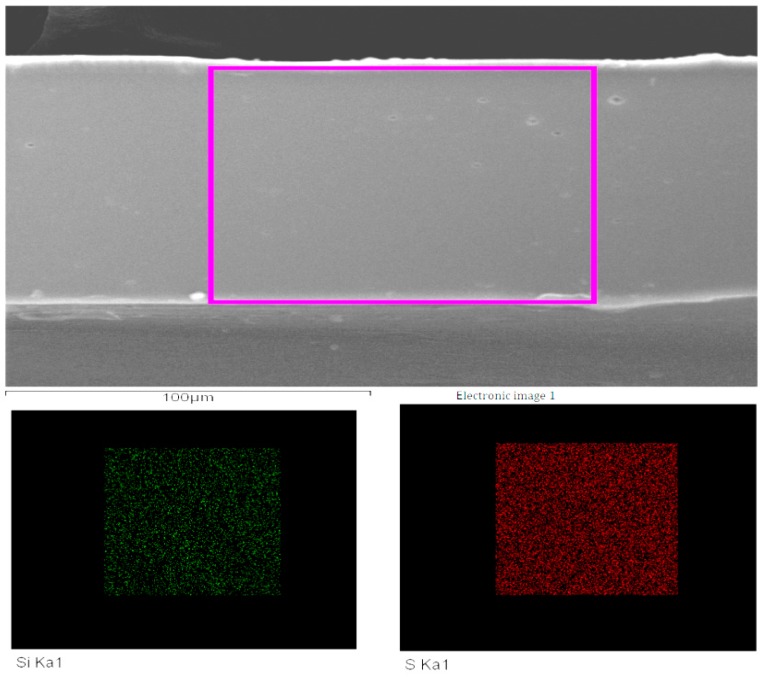
The scanning electron microscopy (SEM) image and EDX distribution maps of Si and S for the cross section of SPEKEBI-2.5-SiO_2_ membrane.

**Figure 5:**
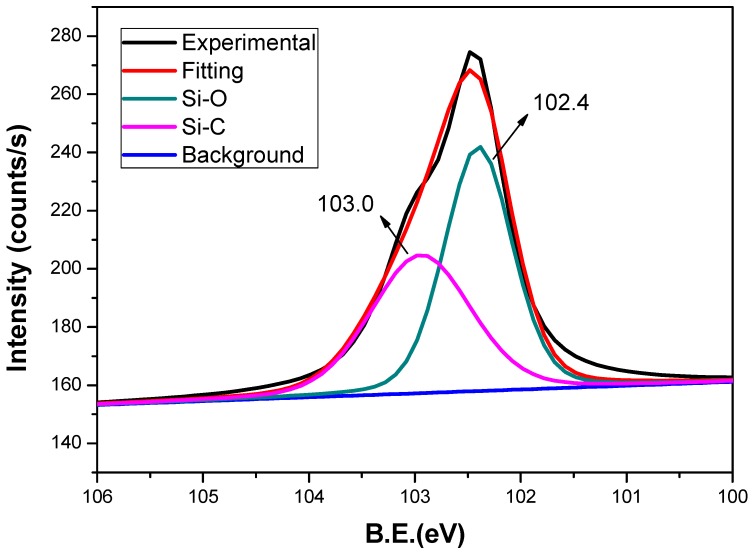
Si 2p XPS spectra for the surface of SPEKEBI-2.5-SiO_2_ membrane.

To gain further confirmatory of the existence of inorganic silica nanoparticles, scanning electron microscopy-energy-dispersive X-ray spectroscopy (SEM-EDS) on the cross-section and XPS on the surface of SPEKEBI-2.5-SiO_2_ were performed as examples. Morphology and elemental distribution for the cross section of SPEKEBI-2.5-SiO_2_ membrane (EDX spectrum and micrograph) are shown in Figure 3 and Figure 4, respectively. It can be seen that the morphology of the membrane is homogeneous and the silicon and sulfur have uniform distributions in the selected section of the membrane. This suggested that the silica networks are distributed evenly in the cross linked membrane. Meanwhile, the XPS spectrum for surface section of SPEKEBI-2.5-SiO_2_ membrane, as shown in Figure 5, also indicated the successful introduction of silica in polymer matrix. The peak at 102.4 eV corresponds to Si-O and Si-C would occur at 103.0 eV [26].

### 3.2. Thermal Stability

**Figure 6:**
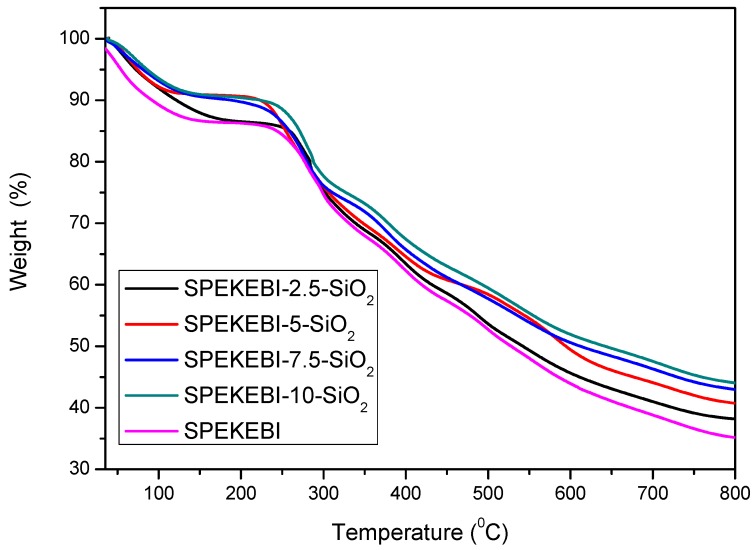
Thermo gravimetric analysis (TGA) diagrams of SPEKEBI-x-SiO_2_ and SPEKEBI membranes.

The thermal stability of SPEKEBI and SPEKEBI-x-SiO_2_ membranes were investigated by thermo gravimetric analysis (TGA), as shown in Figure 6. The TGA analysis revealed that with increasing content of SiO_2_, the weight retention of cross-linked membranes is obviously improved from 33% to 44% at 800 °C. This indicated that the incorporation of inorganic nanoparticles can improve the thermal stability at high temperature.

### 3.3. Mechanical Properties

**Figure 7:**
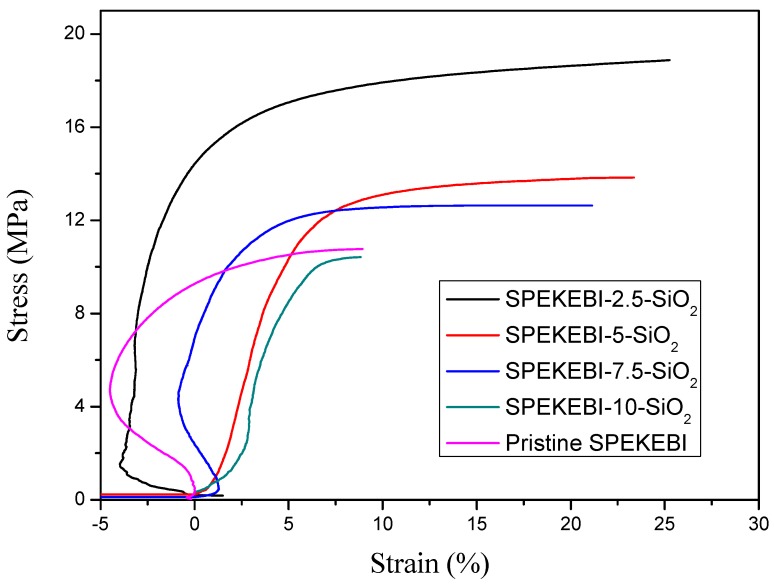
Stress vs. Strain curves of pristine SPEKEBI andSPEKEBI-x-SiO_2_ membranes.

Figure 7 shows the tensile curves of pristine SPEKEBI and SPEKEBI-x-SiO_2_ membranes in a hydrated state at room temperature. The tensile strength values of the SPEKEBI-x-SiO_2_ membranes are in the range of 10.4–18.9 MPa, with the elongation at break of 8.8–25.3%. It can be seen that the SPEKEBI-2.5-SiO_2_ has the highest Eb and Ts values, indicating the dosage control of SiO_2_ in the hybrid membranes is very important. Excessive amounts of SiO_2_ in the polymer matrix may cause phase separation and function in an opposite way. It should be noted that the Eb values of all the covalently cross-linked membranes are larger than 5%, indicating that they possess reasonable toughness despite the occurrence of phase separation.

### 3.4. Oxidative Stability

**Figure 8:**
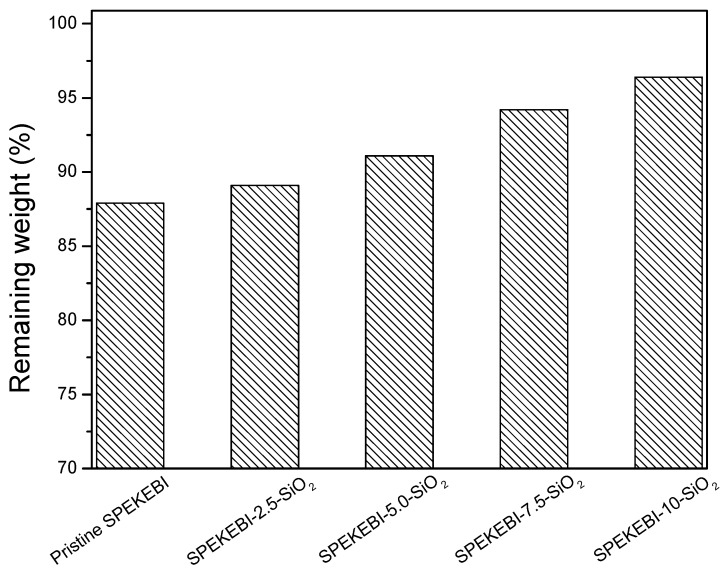
Weight residue of SPEKEBI andSPEKEBI-x-SiO_2_ membranes.

Insufficient lifetime in fuel cell operation is still a key barrier to the widespread commercial use of proton exchange membranes. Oxidative stability has a major influence on the long-term operation of PEMFC systems [17]. In this work, the oxidative stability of membranes was evaluated by immersing the membranes in Fenton’s reagent (3% H_2_O_2_ containing 2 ppm FeSO_4_) at a temperature of 80 °C for 1 h. The weight retention of the membranes was calculated and showed in Figure 8. It was found that the pristine SPEKEBI membrane presented a poorer oxidative stability than the silane cross-linked membranes. What is more, with the increased content of SiO_2_, the oxidative stability of the membranes displayed an increasing trend. These results clearly indicate that the cross-linked network formed between the polymer chains can significantly increase the durability to the oxidizing radical species.

### 3.5. Ion Exchange Capacity, Water Uptake and Swelling Ratio

The water uptake and swelling ratio of PEMs have profound influences on proton conductivity, mechanical strength and dimensional stability [27]. They are both determined by the presence of hydrophilic groups and the membrane structure. In this work, the IECs of the membranes are in a narrow range of 2.21 to 1.82 mmol g^−1^, thus the membrane structure plays a more important role in the determination of the membrane water uptake and swelling ratio. It is well known that high concentration of fixed sulfonic groups will cause the membrane to swell excessively. However, it can be seen from Table 1 that the incorporation of KH-560 had limited the swelling from 159.2% to 106.3% and maintained the water uptake around 30%, which indicated the cross-linked network structure had improved the dimensional stability of the membranes.

### 3.6. Proton Conductivity

**Figure 9:**
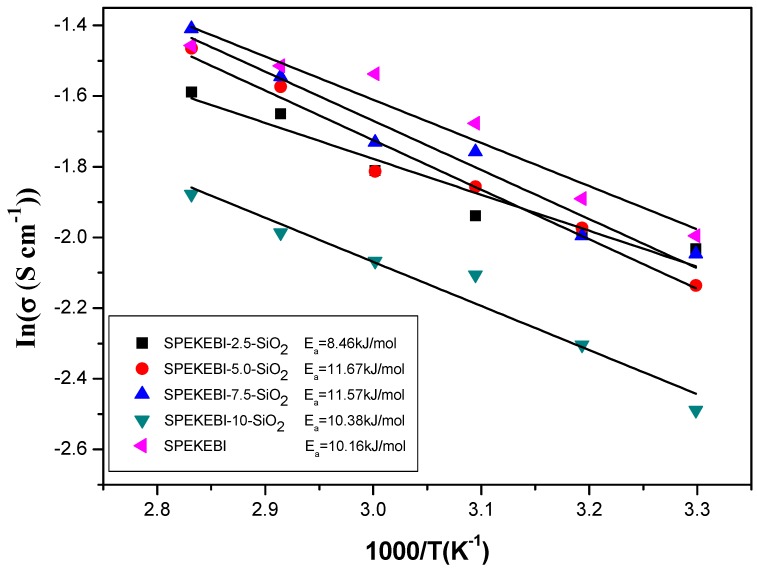
Arrhenius plot of proton conductivity for SPEKEBI-x-SiO_2_ and SPEKEBI membranes.

Proton conductivity is a key factor which determines the efficiency of a fuel cell operation. In this work, the proton conductivity values for the cross-linked membranes in liquid water from 30 °C to 80 °C were measured, and the Arrhenius plots of temperature dependent conductivity are shown in Figure 9. The activation energies (E_a_) were calculated from the slope of the Arrhenius plots to provide insight into the proton conductivity [28]. The introduction of the SiO_2_ decreased the –SO_3_H content in the membranes, which results in the lower IEC as the SiO_2_ content increases from 2.5 wt % to 10 wt % as shown in Table 1. However, the actual changing trend of activation energy is different from the IECs of the membranes for two reasons. Firstly, the –CH_2_CH_2_O– linkages in the cross-linked framework provide bonding sites for hydrogen bonding with water and allow the formation of a bound-water layer which can facilitate the hopping of protons. The second cause is the hydrophobicity of silica framework interrupts the proton transportation though the bound-water layers [19,29,30]. A trade-off exists between these two factors determine the overall proton transfer of the hybrid membranes. The SPEKEBI-2.5-SiO_2_ displayed the lowest E_a_, indicated that the appropriate addition of KH-560 in the SPEKEBI membranes could efficiently enhanced the proton conductivity. Meantime, it should be noted that the SPEKEBI-7.5-SiO_2_ membrane showed higher proton conductivity than the pristine SPEKEBI membrane in high temperature (at 80 °C, 244 ms cm^−1^) which should be attributed to the low swelling effect of the hybrid membranes.

### 3.7. Methanol Permeability and Selectivity

**Figure 10:**
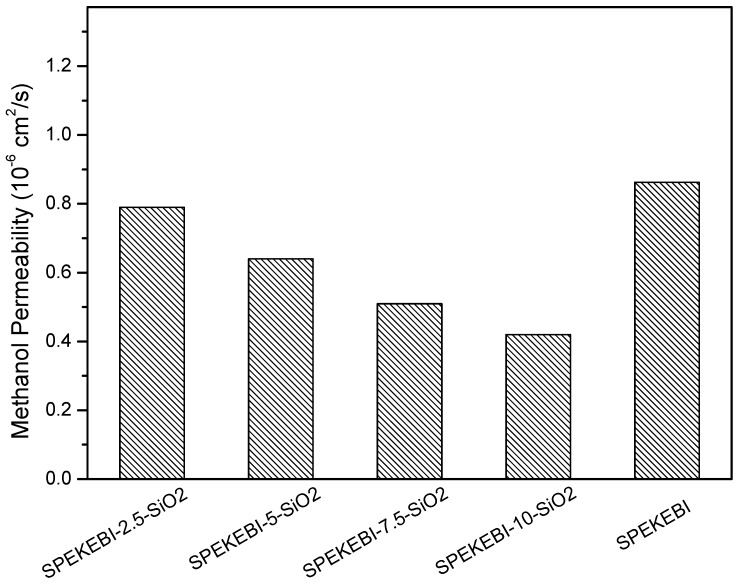
Methanol permeability of SPEKEBI-x-SiO_2_ and SPEKEBI membranes.

For a successful PEM, excellent methanol resistance is a necessary requirement. As reported in our prior work [31,32], the acid-base interactions can act as a barrier for methanol crossover. However, the SPEKEBI polymer has a very high sulfonation degree and the density of sulfonated hydrophilic groups is much higher than the density of basic imidazole repeat units. Besides, the acid-base interactions may affect the proton conductivity of the membranes. Therefore, the introduction of the SiO_2_ network is a better approach to control the methanol permeability. As shown in Figure 10, the methanol permeability decreases with increasing SiO_2_ content for the cross-linked membranes. All the hybrid membranes showed a better methanol blocking effect than the pristine SPEKEBI membrane. This could be attributed that SiO_2_ hydrophobic cross-linking networks increased the length of paths along which methanol is transported.

In fuel cell applications, high proton conductivity always causes severe fuel leakage. Thus, the selectivity, defined as the ratio of proton conductivity to methanol permeability, is also an important parameter to estimate the membrane performance in DMFC. As shown in Table 2, the selectivity was increased with the loading amount of KH-560. However, when the SiO_2_ content reached to 10%, the selectivity showed a little decrease, since the decrease of methanol permeability is less predominant than the decrease of proton conductivity.

### 3.8. Single Fuel Cell Performance

**Figure 11:**
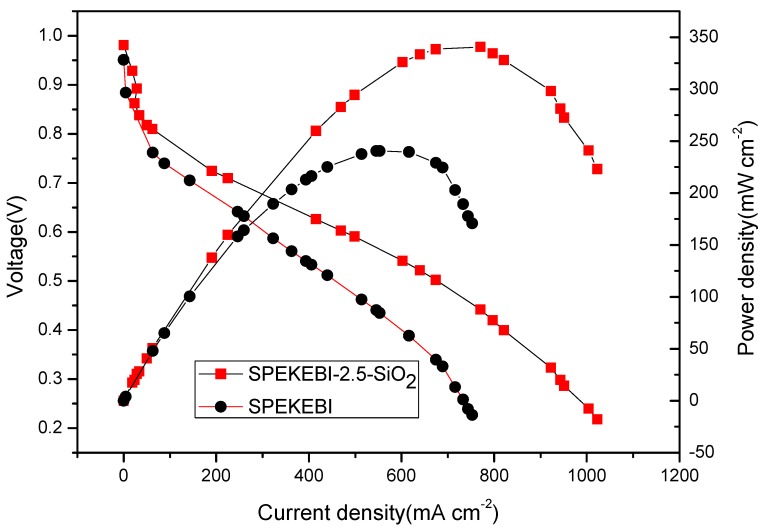
Single fuel cell performance of SPEKEBI-2.5-SiO_2_ and SPEKEBI membranes operating under 100% RH at 75 °C.

In order to further confirm the improvement effect of KH-560 on electrochemical performance of SPEKEBI, single fuel cell tests were applied with 100% RH humidified H_2_/O_2_ at 75 °C. With the mechanical property and proton conductivity taken into consideration, the composite membrane SPEKEBI-2.5-SiO_2_ and pure SPEKEBI were chosen to assemble single fuel cells. As shown in Figure 11, the open-circuit voltages (OCVs) for the two membranes are more than 0.96 V, which indicated that the membranes are pore-free and exhibit low gas crossover. The maximum power density of SPEKEBI-2.5-SiO_2_ membrane was 340.6 mW cm^−2^, while the pure SPEKEBI membrane displayed a maximum power density of 240.5 mW cm^−2^. Obviously, the crosslinked membrane showed a better single cell performance than that of pure SPEKEBI membrane.

## 4. Conclusions

Highly stable silane cross-linked side-chain-type sulfonated poly(ether ketone/ether benzimidazole) with high ion exchange capacities was successfully synthesized. The SPEKEBI polymer with densely sulfonated segments was reacted with KH-560 by a heating method at a comparatively low temperature. After that, silane cross-linked structures were formed in the membranes by in situ sol-gel process in hydrochloric acid solution. The resulting SPEKEBI-x-SiO_2_ membranes showed well-developed stability and selectivity compared to the pristine SPEKEBI membranes. As observed by the SEM-EDS measurement, the SiO_2_ nanoparticles had a uniform distribution and no phase separation appeared in the membrane. The SPEKEBI-7.5-SiO_2_ membrane possessed higher proton conductivity at 80 °C than the pristine SPEKEBI membrane, which is attributed to a much smaller swelling degree in high temperature. To sum up, it appears that the SPEKEBI-x-SiO_2_ membranes showed high thermal and dimensional stability, acceptable proton conductivity and enhanced relative selectivity, which makes the silane cross-linked membranes very attractive for fuel cell application as potential PEMs. Besides, a single H_2_/O_2_ fuel cell equipped with the crosslinked membrane showed a maximum power density of 340.6 mW cm^−2^, which is much higher than that of pure SPEKEBI membrane under the same conditions. These results suggested that silane crosslinked composites possess strong potential to serve as high performance polymer electrolyte membrane materials.

## Figures and Tables

**Table 1:** Ion-exchange capacities (IECs), water uptake and swelling ratio of SPEKEBI-x-SiO_2_ and SPEKEBI membranes.

Membranes	IEC		Water Uptake (%)		Swelling Ratio (%)	
	Theoratical	Practical	30 °C	80 °C	30 °C	80 °C
SPEKEBI-2.5-SiO_2_	2.30	2.21	42.8	159.2	8.9	44.8
SPEKEBI-5.0-SiO_2_	2.24	2.09	37.7	128.0	6.4	31.8
SPEKEBI-7.5-SiO_2_	2.18	1.99	37.0	118.9	5.0	29.0
SPEKEBI-10-SiO_2_	2.12	1.82	30.8	106.3	4.8	28.6

**Table 2:** Selectivity of SPEKEBI-x-SiO_2_ and SPEKEBI membranes.

Membranes	б (mS cm^−1^)		P_MeOH_	Selectivity
	30 °C	80 °C	(×10^−7^ cm^2^ s^−1^)	(×10^−4^ S s cm^−3^)
SPEKEBI	136	233	8.62	15.8
SPEKEBI-2.5-SiO_2_	131	204	7.91	16.6
SPEKEBI-5.0-SiO_2_	118	231	6.54	18.0
SPEKEBI-7.5-SiO_2_	129	244	5.81	22.2
SPEKEBI-10-SiO_2_	83	153	4.22	19.7
